# A modification of nested PCR method for detection of *Enterocytozoon hepatopenaei* (EHP) in giant freshwater prawn *Macrobrachium rosenbergii*

**DOI:** 10.3389/fcimb.2022.1013016

**Published:** 2022-09-23

**Authors:** Yuan Wang, Jinyang Zhou, Menghe Yin, Na Ying, Yang Xiang, Wenchang Liu, Junqiang Ye, Xincang Li, Wenhong Fang, Hongxin Tan

**Affiliations:** ^1^ Key Laboratory of East China Sea Fishery Resources Exploitation, Ministry of Agriculture and Rural Affair, East China Sea Fisheries Research Institute, Chinese Academy of Fishery Sciences, Shanghai, China; ^2^ Shanghai Engineering Research Center of Aquaculture, Shanghai Ocean University, Shanghai, China; ^3^ Shanghai Collaborative Innovation Center for Cultivating Elite Breeds and Green-culture of Aquaculture Animals, Shanghai, China; ^4^ Fisheries Technology Promotion Station of Fengxian District, Shanghai, China

**Keywords:** microsporidia, *Enterocytozoon hepatopenaei*, *Macrobrachium rosenbergii*, nested PCR, spore wall protein

## Abstract

The microsporidian *Enterocytozoon hepatopenaei* (EHP) has become a critical threat to the global shrimp aquaculture industry, thus necessitating early detection by screening. Development of a rapid and accurate assay is crucial both for the active surveillance and for the assessment of shrimp with EHP infection. In the present study, a distinct strain of *E*. *hepatopenaei* (EHP*_Mr_*) was found in *Macrobrachium rosenbergii*. The SWP1 gene analysis revealed it was a new genotype that differed with the common strain isolated from the *Litopenaeus vannamei* (EHP*_Lv_*). A nested SWP-PCR method was modified to fix the bug that the original inner primers could not recognize the EHP*_Mr_* strain. The redesigned inner primers successfully amplified a product of 182 bp for both the EHP*_Mr_* strain and the EHP*_Lv_* strain. The new primers also had good specificity and high sensitivity, which may serve as an alternative for EHP genotyping. This study provided a method for detection of EHP in the biosecurity of *Macrobrachium rosenbergii* farming, and the developed protocol was proposed for the routine investigation and potential carrier screening, especially for molecular epidemiology.

## 1 Introduction

*Enterocytozoon hepatopenaei* (EHP) is a microsporidian responsible for hepatopancreatic microsporidiosis (HPM) outbreaks in cultured shrimp (Tourtip et al., 2009; Chaijarasphong et al., 2021). In recent years, EHP has been discovered in several countries, such as Malaysia, Vietnam, India, Indonesia, Thailand, China, and Venezuela, and it has caused huge economic losses (Biju et al., 2016; Tang et al., 2017; Flegel, 2018; Behera et al., 2019; Hou et al., 2021). Its widespread distribution has increased the threat to the global shrimp aquaculture industry. The maintenance of shrimp broodstock and the management of the hatchery, nursery and grow-out are facing enormous challenges in the prevention and control of EHP disease.

The giant freshwater prawn, *Macrobrachium rosenbergii*, native to the tropical and subtropical areas of Southeast Asia, is one of the important economic prawn species in the world (Azad et al., 2021). It was introduced into China from Japan in 1976 (Dong et al., 2020). Being popular for its large individual size, fast growth, delicious flesh and high nutritional value, it has become one of the important cultured species in China (Wei et al., 2021). Shrimp hosts known to be infected by EHP include *P. monodon*, *L. vannamei*, *Litopenaeus stylirostris*, and a suspected species (*Penaeus japonicus*) (Chaijarasphong et al., 2021), but there are few reports about *M. rosenbergii* being infected by EHP.

Specific pathogens screening and detection from the shrimp postlarvae stages have become the important measures taken by farmers to ensure the success of aquaculture. The nested PCR diagnostic technique is widely used because of its high accuracy and low instrument requirements. However, the accuracy and sensitivity of the nested PCR assays established based on different gene sequences are different. Previous research has confirmed that the primers designed based on the EHP SSU rRNA gene can cross-react with other similar microsporidians and generate false positives, but the primers designed based on the spore wall protein (SWP) gene can avoid false positives and are more sensitive than the former (Jaroenlak et al., 2016). Therefore, the nested PCR assay targeting the SWP gene (SWP-PCR) has been adopted by the fishery industry and widely used in the detection of shrimp seedlings. Furthermore, this method has also been selected as the EHP detection standard for the fishery industry in China (SC/T 7232-2020 code of diagnosis for *Enterocytozoon hepatopenaei* disease).

In March 2020, the above SWP-PCR method was applied by our laboratory to screen for pathogens in *M. rosenbergii* seedlings. Interestingly, we found that the positive target fragment did amplify by the outer primers, but no band was amplified by the inner primers. In subsequent studies, we confirmed the EHP strain derived from *L. vannamei* (EHP*_Lv_*) and the EHP strain derived from *M. rosenbergii* (EHP*_Mr_*) were different in SWP gene, although the SSU rDNA sequence of these two EHP strains showed ~99% identity. The above SWP-PCR method was not suitable for the detection of EHP*_Mr_*. The goal of this study was to develop a sensitive and specific nested PCR method for simultaneous detection of two EHP strains.

## 2 Material and methods

### 2.1 Samples collection

The EHP-infected *L. vannamei* and the EHP-infected *M. rosenbergii* were collected from the same farm in Fengxian District, Shanghai Province, China (N30°53’18.6”, E121°35’32.3”), in March 2020. The farm suffered severe EHP infection in 2019. The infected *L. vannamei* were 2.0 ~ 3.0 cm in length. The unnormal *M. rosenbergii* were 1.0 ~ 1.8 cm in length. Both healthy *L. vannamei* and healthy *M. rosenbergii* were collected from the normal ponds on another farm in Fengxian District. Samples were transported to the laboratory with oxygen and then fixed in 95% ethanol for PCR analysis. The handling of shrimps followed the guidelines for the Ethical Committee of Experimental Animal Care at the Shanghai Ocean University of China.

### 2.2 DNA extraction

For EHP detection, 10 individual samples of each shrimp and prawn were dissected. The hepatopancreas DNA was extracted using an animal organization DNA Extraction Kit (Tiangen Biotechnology, China) according to the manufacturer’s instructions, and stored at −20°C for PCR assays.

### 2.3 SWP1 gene amplification and cloning

The target genes were amplified using the nested SWP-PCR method. Briefly: the PCR mixtures (25 μL) for both steps contained 0.625 units of Ex Taq DNA polymerase (Takara Bio) and 0.2 μM of each primer. For the first PCR reaction, outer primers SWP1F and SWP1R (**Table 1**) were used to amplify a 514 bp fragment. The PCR cycling conditions include an initial denaturation at 95°C for 5 min, followed by 30 cycles of 95°C for 30 s, 58°C for 30 s, and 68°C for 45 s, and a final extension at 68°C for 5 min. The PCR products were checked using 1% agarose gel with DNA ladder.

**Table 1:** Primers for PCR method.

Primer Name	Sequence (5′-3′)	Site	Amplicon size (bp)	References
SWP1F	TTGCAGAGTGTTGTTAAGGGTTT	130	514	Jaroenlak et al., 2016
SWP1R	CACGATGTGTCTTTGCAATTTTC	643		
SWP2F	TTGGCGGCACAATTCTCAAACA	167	147	
SWP2R	GCTGTTTGTCTCCAACTGTATTTGA	313		
SWP2F′	GCAGAGTGTTGTTAAGGGTTTAAG	132	182	This study
SWP2R′	GCTGTTTGTCWCCAACTGTATT	313		

the site is based on the reference sequence (GenBank accession nos. **MG015710**).

The DNA fragments of the SWP1 gene from both EHP isolates were purified and cloned into the pMD18-T vector. The resulting plasmids were transferred into competent cells DH5α and cultured in Luria-Bertani (LB) medium. Recombinant colonies were selected by the Blue-White screening. The transformants were identified by colony PCR and then cultured in a shaking incubator for 4 hours. Finally, the positive colonies were sequenced using M13 sequencing primers by the ABI 3730xl DNA Analyzer.

### 2.4 Sequence and phylogenetic analysis of SWP1 gene

For sequence homology analysis, the full-length SWP1 gene (EhSWP1, GenBank accession nos. MG015710, GenPept accession nos. AVQ09707) previously published by Jaroenlak et al. (2018) was used as the reference sequence, which was amplified from EHP strain isolated from *P*. *vannamei*. After cloning and sequencing, the nucleotide sequences obtained were edited and alignments performed using Clustal W and compared with other nucleotide sequences in the GenBank using the BLAST program at National Center for Biotechnology Information (NCBI).

For the SWP1 gene phylogeny, multiple nucleotide sequence alignment was carried out using Clustal W. The “find best DNA/Protein models” program was run to determine the best-fit model with the lowest Bayesian information criterion (BIC). Phylogenetic tree was constructed using Neighbor-Joining method based on the Tamura 3-parameter model in MEGA-X. Bootstrap with 1000 replications was set to assess branch support.

### 2.5 New inner primers designed for simultaneous detection

To solve the problem that the primers SWP2F and SWP2R could not amplify the SWP1 gene of EHP*_Mr_*, the new inner primer pairs were designed with the aid of Primer Premier 6.0 software. Based on multiple sequence alignments results, primer positions were derived from the conserved regions of SWP1 gene from all EHP isolates. Specificity of the primers was initially checked using primer blast (www.ncbi.nlm.nih.gov/tools/primer-blast/). The new inner primer pairs, forward primer SWP2F′ (5’-GCAGAGTGTTGTTAAGGGTTTAAG-3’) and reverse primer SWP2R′ (5’-GCTGTTTGTCWCCAACTGTATT-3’), were designed to target 182 bp internally to the first PCR product (**Table 1**).

### 2.6 Comparison of original method and modified method for detection of two EHP isolates

To compare the validity of newly designed inner primers and the original inner primers, the external PCR products of three SWP-PCR positive *L. vannamei* (EHP*_Lv_*) and three SWP-PCR positive *M. rosenbergii* (EHP*_Mr_*) were selected as DNA template respectively.

For the original SWP-PCR method, the inner primers SWP2F and SWP2R (**Table 1**) were used to generate a 147 bp fragment. The thermal cycling conditions include an initial denaturation at 95°C for 5 min, followed by 20 cycles of 95°C for 20 s, 64°C for 30 s, and 68°C for 20 s, and a final extension at 68°C for 5 min (Jaroenlak et al., 2016).

For the modified SWP-PCR method, 2nd-step (nested) PCR was carried out with the inner primers SWP2F′ and SWP2R′ (**Table 1**) to amplify a 182 bp product. PCR cycling conditions were initiation denaturation at 95°C for 5 min, followed by 20 cycles of 95°C for 30 s, 55°C for 30 s, and 68°C for 20 s, and a final extension at 68°C for 5 min. All secondary PCR products were analyzed by electrophoresis on a 1% agarose gel.

### 2.7 Sensitivity of the modified nested PCR assay

The plasmid containing the SWP1 gene of the EHP*_Lv_* (named pGEM-SWP1) was extracted from the positive colonies as described above. The series of 10-fold dilutions of pGEM-SWP1 were used as positive templates. The single PCR with the two primers sets SWP2F/2R and SWP2F′/2R′ were carried out respectively, for testing the comparative sensitivity of the modified nested PCR and original nested PCR.

### 2.8 Specificity of the modified nested PCR assay

The genomic DNAs of five different aquatic microsporidians were selected to evaluate the specificity of the designed inner primers. *Enterospora epinepheli* isolated from *Epinephelus* spp.; *Nucleaspora hippocampi* isolated from *Hippocampus erectus* (Wang et al., 2022); *Enterocytospora artemiae* isolated from *Palaemonetes sinensis*; *Ameson portunus* isolated from *Portunus trituberculatus*; *Potaspora* sp. (unidentified) isolated from *Exopalaemon carinicauda*. *Enterospora* and *Nucleaspora* were the closely related genus in the *Enterocytozoon* group Microsporidia (EGM) that mainly infect gastrointestinal tracts of their hosts (Stentiford et al., 2019). *E. artemiae* infected the hepatopancreas and gut of crustacean hosts (Rode et al., 2013). *Ameson* and *Potaspora* were selected as the representative species of microsporidia infecting the skeletal muscles of hosts (Ding et al., 2016; Wang et al., 2017). The DNA template extracted from EHP*_Mr_*-infected *M. rosenbergii* was used as a positive control. The nested PCR conditions referred to the above.

## 3 Results

### 3.1 Comparison of SWP genes between two strains of EHP

#### 3.1.1 Nucleic acid sequence analysis

**Figure 1:**
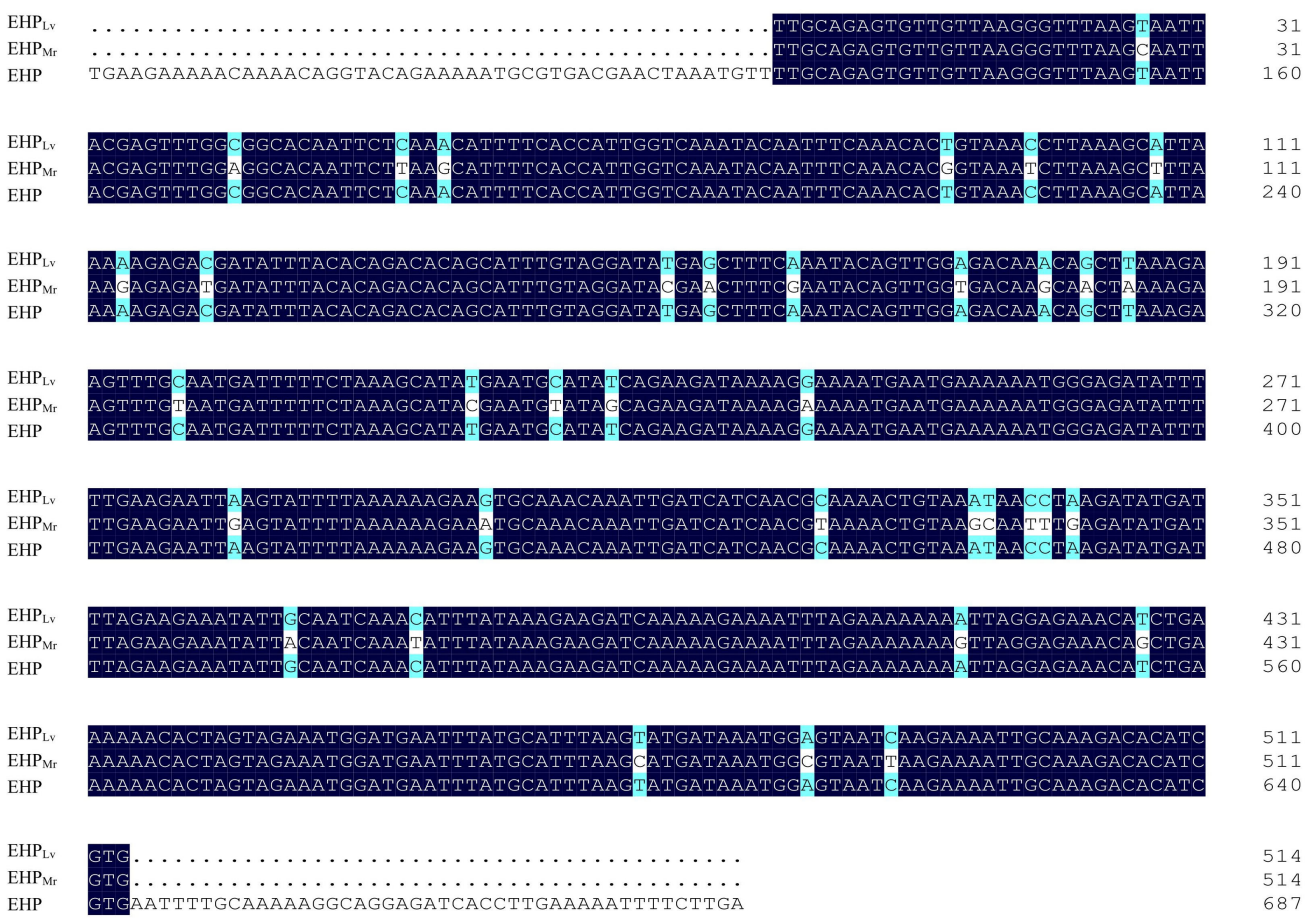
Alignment of partial nucleotide sequences of the SWP1 gene of EHP*_Lv_*, EHP*_Mr_*, and reference EHP (MG015710). The nucleobases with white differ from the consensus. The numbers on the right indicate the nucleotide position.

The obtained partial SWP1 gene of EHP*_Lv_* (Eh*_Lv_* SWP1) and the partial SWP1 gene of EHP*_Mr_* (Eh*_Mr_* SWP1) were both 514 bp in size (**Figure 1**). Sequence analysis revealed that the Eh*_Lv_* SWP1 shared a 100% nucleotide sequence identity with the reference EhSWP1. Whereas the Eh*_Mr_* SWP1 shared a 93% nucleotide sequence identity with the EhSWP1. This indicated that the Eh*_Mr_* SWP1 represented the presence of a novel genotype. In comparison with Eh*_Lv_* SWP1 and EhSWP1, the Eh*_Mr_* SWP1 showed 36 single base mutations, including eight transitions and 28 transversions, and no insertion and deletion (**Figure 1**). The obtained nucleotide sequence of Eh*_Mr_* SWP1 was deposited in GenBank database under accession number **MW269619**.

#### 3.1.2 Amino acid sequence comparison

**Figure 2:**
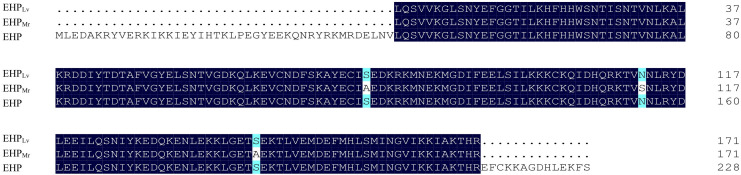
Alignment of partial protein sequences of the SWP1 from EHP*_Lv_*, EHP*_Mr_*, and reference EHP (GenPept accession nos. AVQ09707). The amino acids with white differ from the consensus. The numbers on the right indicate the amino acid position in the published sequence.

The comparison of predicted protein sequences revealed that EHP*_Lv_* shared a 100% identity with the reference EHP from *L. vannamei* (GenPept accession nos. AVQ09707), whereas EHP*_Mr_* shared a 98.25% amino acid sequence identity to the reference EHP (**Figure 2**). Remarkably, among the obtained 171 amino acids, three amino acid exchanges: serine mutated to alanine at sites 77 and 143, and asparagine mutated to serine at site 112.

#### 3.1.3 Phylogenetic analyses

**Figure 3:**
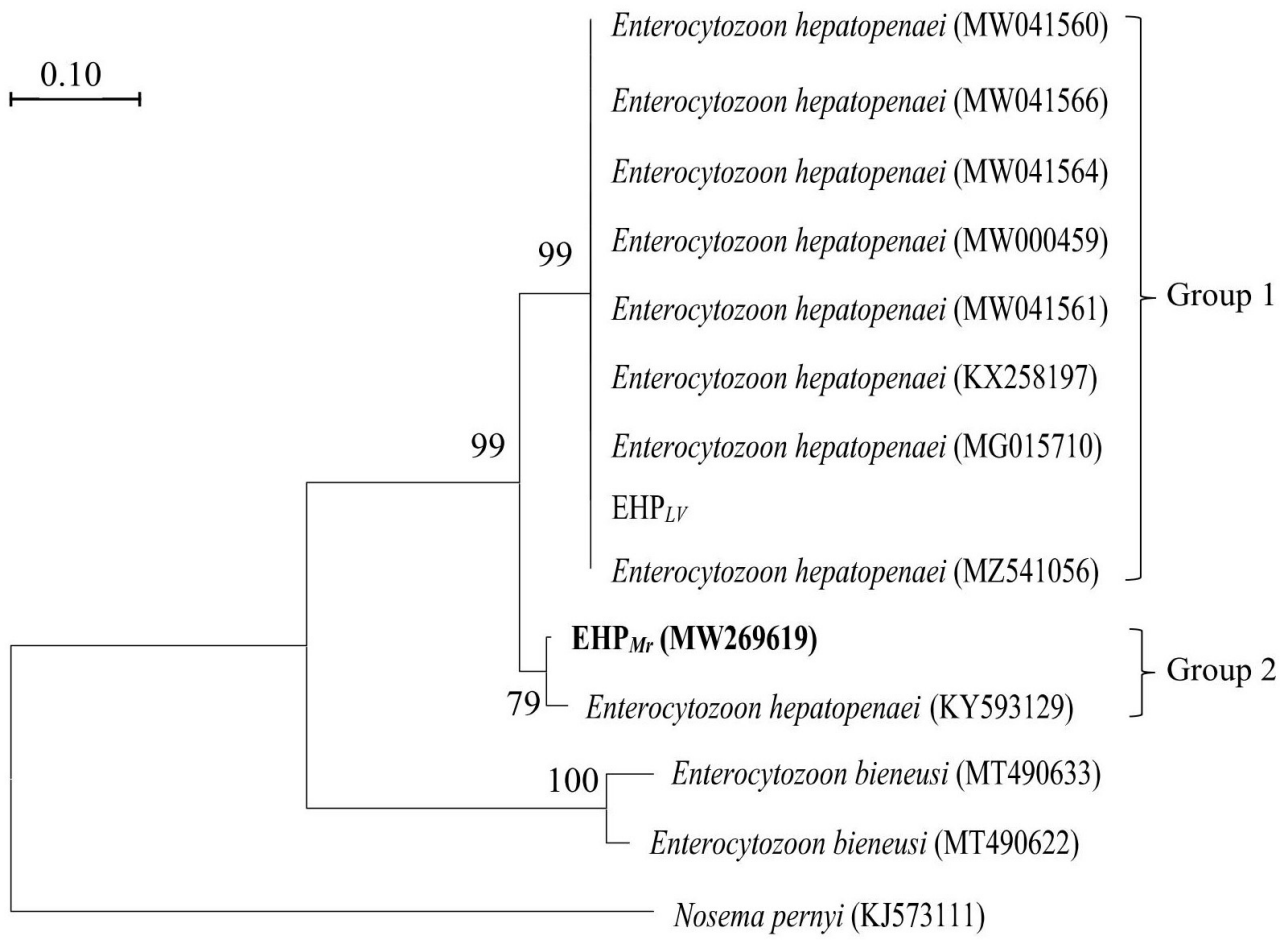
Phylogenetic tree of SWP1 gene of EHP isolates with other microsporidia. *Nosema pernyi* is used as the outgroup. Bootstrap values are indicated on the branches.

On the phylogenetic tree (**Figure 3**), all EHP isolated were grouped together in a large branch with a high support (99%). Group 1 is largest group containing most EHP that infecting the *L. vannamei.* Within Group 2, EHP*_Mr_* clustered together with an EHP strain obtained from *L. vannamei* (KY593129).

### 3.2 Detection of EHP*_Lv_* and EHP*_Mr_* by the existing SWP-PCR and the modified SWP-PCR

**Figure 4:**
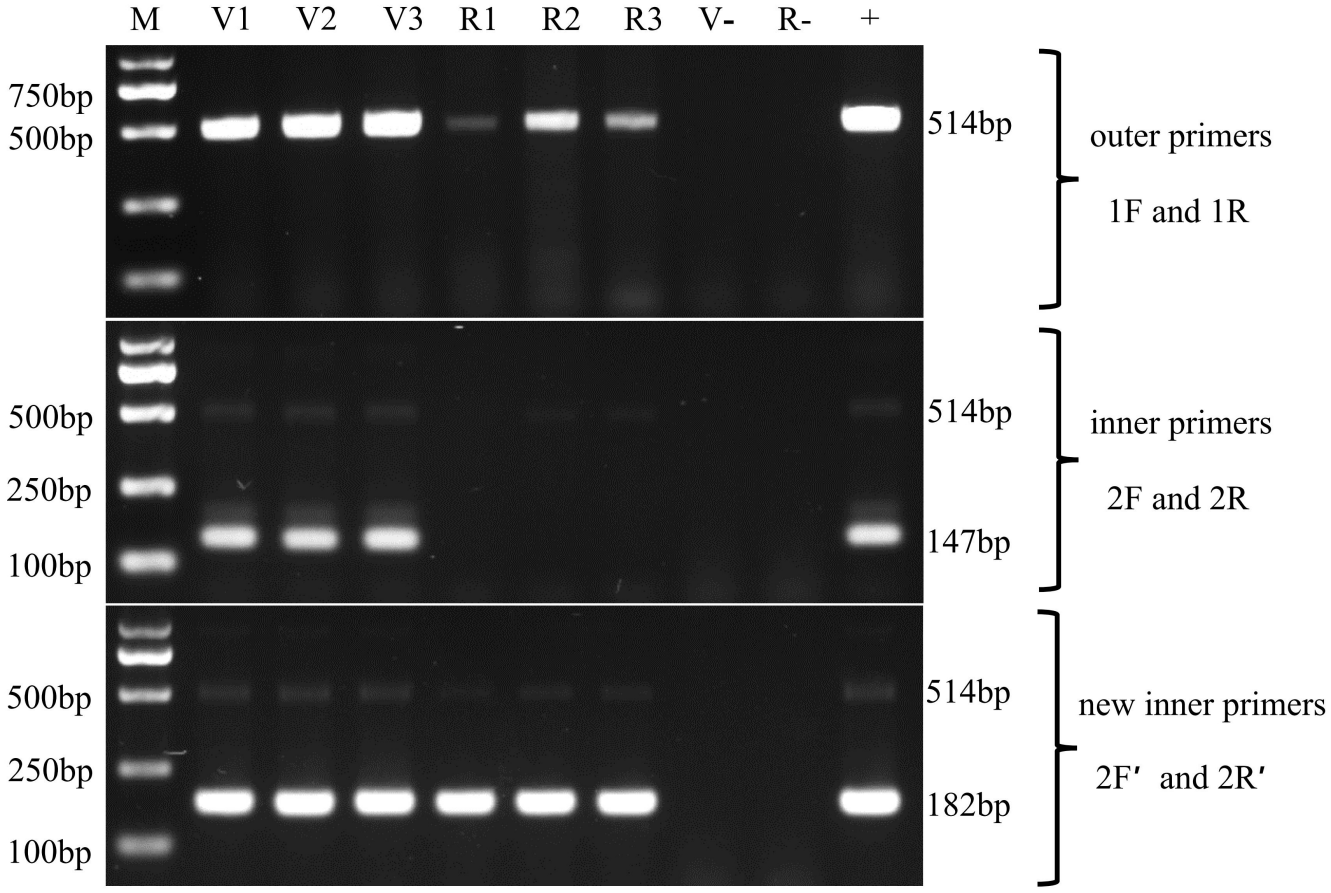
Nested PCR for the detection of the two EHP stains in *Litopenaeus vannamei* and *Macrobrachium rosenbergii* by using the SWP2F/2R primers and SWP2F′/2R′ primers, respectively. Lanes V1~V3: the hepatopancreatic DNA of EHP*_Lv_*-infected *Litopenaeus vannamei*; Lanes R1~R3: the hepatopancreatic DNA of EHP*_Mr_*-infected *Macrobrachium rosenbergii*; M: molecular weight marker; +: positive control, SWP1 gene plasmid DNA; v-: negative control, the hepatopancreatic DNA of healthy *Litopenaeus vannamei*, R-: negative control, the hepatopancreatic DNA of healthy *Macrobrachium rosenbergii*.

To validate the two protocols, the hepatopancreas DNAs, isolated from naturally EHP*_Lv_*-infected *L. vannamei* samples and naturally EHP*_Mr_*-infected *M. rosenbergii* samples, were subjected to the first round of amplification. The outer primers SWP1F/1R successfully amplified a 514 bp DNA fragment from both infected *L. vannamei and* infected *M. rosenbergii* (**Figure 4**, top).

In the second round, the inner primers SWP2F/2R amplified the expected 147 bp fragment from all EHP*_Lv_*-infected *L. vannamei* but were negative for any of the EHP*_Mr_*-infected *M. rosenbergii* (**Figure 4**, middle), it means the existing SWP-PCR method can only detect the EHP*_Lv_*. Whereas the novel inner primers SWP2F′/2R′ produced the predicted 182 bp fragment for both infected shrimp and infected prawn (**Figure 4**, bottom), indicating that the modified SWP-PCR method can detect not only EHP*_Lv_* but also EHP*_Mr_*.

In addition, comparing the second-round PCR products by the two methods, the typical products of the existing SWP-PCR method contained an unexpected DNA fragment which migrated very closely together with the target band of 147 bp (**Figure 4**, middle), similar to previous reports (Jaroenlak et al., 2016; Munkongwongsiri et al., 2022). While only one prominent band of 182 bp was formed by the novel primers, indicating that the modified method improved the specificity of PCR amplification (**Figure 4**, bottom).

### 3.3 Sensitivity of the nested PCR

**Figure 5:**
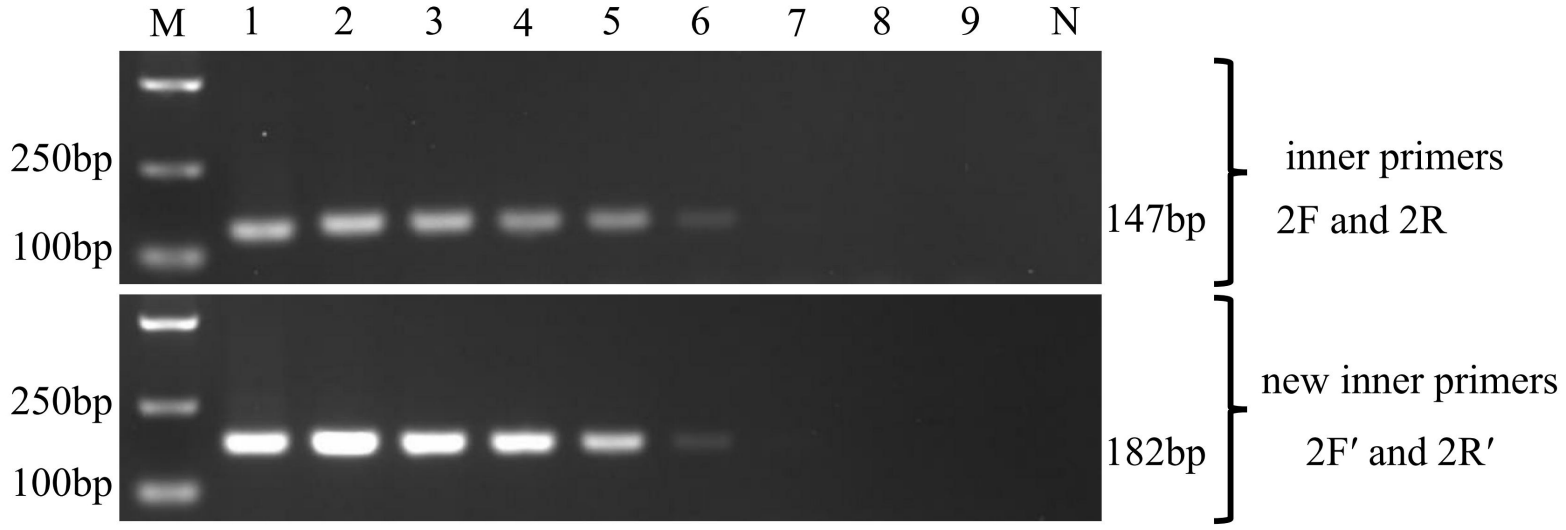
Comparison of sensitivity of the inner primers (SWP2F/2R) and new inner primers (SWP2F′/2R′) to amplify the SWP1 gene. M: molecular weight marker; 1-9:1×10^8^ -1×10^0^ copies of 10-fold dilutions of pGEM-SWP1; N: negative control, DNA samples of healthy *P*. *vannamei*.

The sensitivity of the two nested PCR was tested using the 10-fold dilution series of pGEM-SWP1 plasmid DNA. The result was shown in **Figure 5**. In the single PCR, the modified method displayed a high sensitivity identical to the original SWP-PCR method, which could detect as low as 10^3^ copies of pGEM-SWP1 per reaction mix.

### 3.4 Specificity of the nested PCR

**Figure 6:**
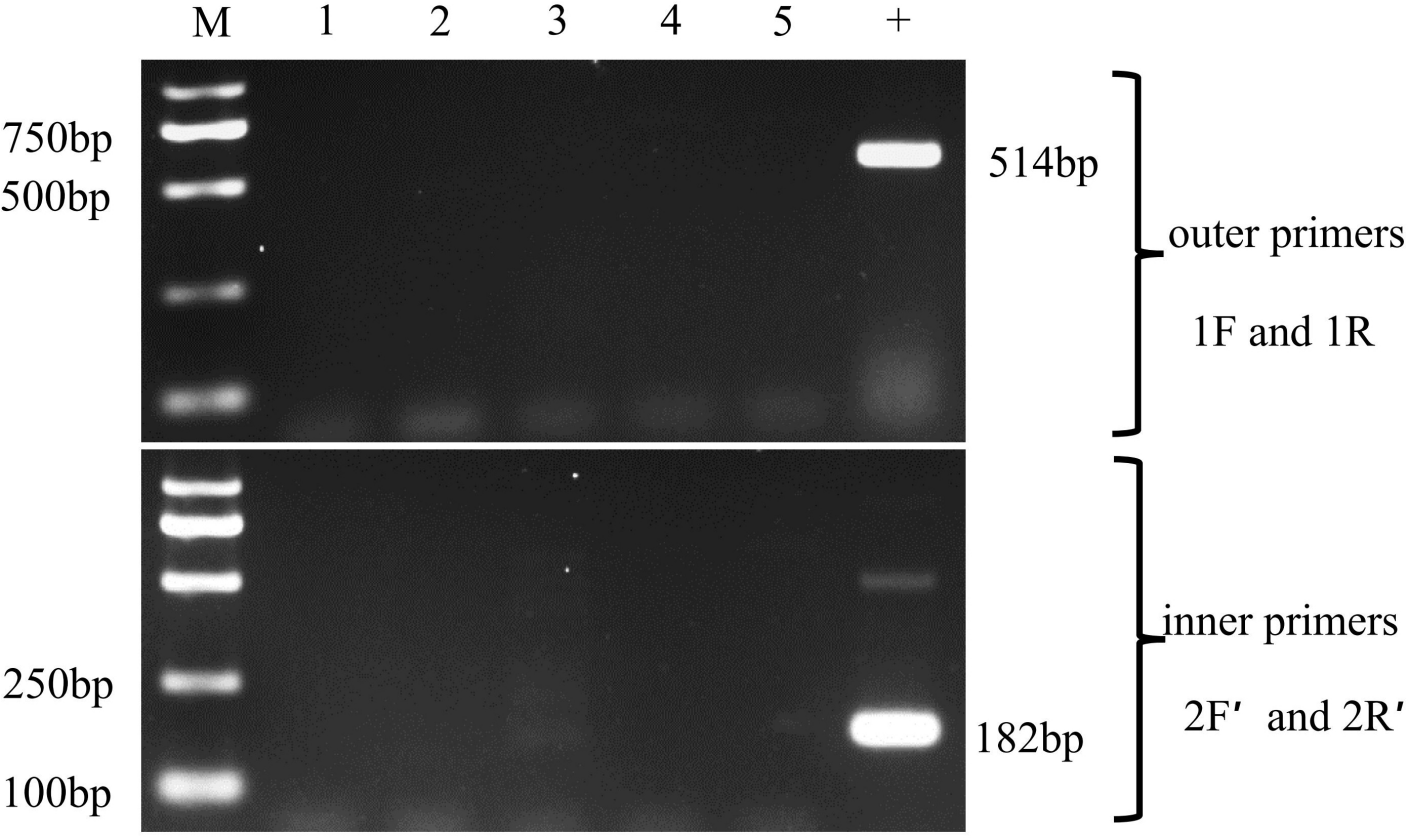
Validation of the specificity of improved nested PCR detection. M: molecular weight marker; lane 1, *Enterospora epinepheli*; lane 2, *Nucleaspora hippocampi*; lane 3, *Enterocytospora artemiae*; lane 4, *Ameson portunus*; lane 5, *Potaspora* sp.; +: positive control.

In cross-amplification assays, none of the other microsporidian showed any amplification product in the nested PCR (**Figure 6**). This confirmed the specificity of the designed primers for EHP detection.

## 4 Discussion

### 4.1 The application of new nested PCR for discovering the EHP mutants

Microsporidia are known as the obligate intracellular parasite. To adapt to the host cell life, its genome has been extremely compressed (Corradi and Slamovits, 2011). The natural genetic variation of pathogenic microorganisms can determine the success of infecting the host and favorable mutations may help to expand its host range (Bonneaud and Longdon, 2020). Variants have been found in a variety of microsporidia, such as *Encephalitozoon cuniculi*, *Encephalitozoon hellem*, *Encephalitozoon intestinalis* and *Enterocytozoon bieneusi* (Duzlu et al., 2019; Li et al., 2019). According to the results of this study, a mutant strain of EHP has been detected in *M. rosenbergii* for the first time, and the difference of its SWP1 gene suggested that EHP is also quietly changing itself to infect other crustacean hosts.

The nested PCR method has become the common method for disease surveillance because of its high specificity and sensitivity. However, due to the strain differentiation of EHP, the single specific primers cannot recognize different strains of EHP. To solve this problem, in this study, we designed a pair of degenerate primers to meet the demand for the EHP mutants’ detection in disease control and prevention.

Furthermore, the combined use of SWP2F′/2R′ primer pairs and SWP2F/2R primer pairs will help us to identify EHP*_Lv_* and EHP*_Mr_* strains. In a batch of shrimp infected with EHP, if SWP2F′/2R′ is positive and SWP2F/2R test is negative, it suggests that this batch of samples is infected with EHP*_Mr_* strain; if SWP2F′/2R′ is positive and SWP2F/2R test is also positive, it shows that this batch of samples is infected with EHP*_Lv_* strain.

### 4.2 SWP1 gene can be a recognizing site for EHP genotyping

Most researches on genotyping of microsporidia mutant strains use ITS site as diagnostic target and seldom use SWP gene. However, in recent years, more and more studies demonstrated that SWP gene was a promising target for genotyping. Xiao et al. (2001) found that the SWP1 gene of *E. cuniculi* had genetic diversity; Ou et al. (2021) confirmed the canine-adapted genotypes (Group 11) of *E. bieneusi* are one unique group of genotypes, and genetically divergent from other genotype groups by the sequence difference in SWP1 gene. Polonais et al. (2010) found that the EhSWP1 C-terminal of four strains of human microsporidia *E. hellem* showed significant interspecific and intraspecific polymorphisms, suggesting that SWP gene is more suitable for genotyping than internal transcribed spacer (ITS) or small subunit ribosomal DNA (SSU-rDNA) sequences.

To investigate the spread of EHP in global shrimp aquaculture, genotyping will become an important problem that needs to be solved in epidemiology. The polymorphism of SWP1 gene in different isolates will make it a good marker for studying EHP genotyping. In addition, the mutation of SWP1 gene may help to increase our understanding of the adhesion of EHP spore wall proteins to different host cell surface receptors.

## 5 Conclusion

In summary, it is the first report on characterization of the SWP1 gene from a new EHP genotype. The mutation of the SWP1 gene will be useful to understand the molecular mechanism that EHP adapts to different hosts. Furthermore, this study provides a modified nested PCR assay for EHP detection in both *L. vannamei* and *M. rosenbergii*. The modified method possesses excellent specificity and comparable sensitivity with the previous nested PCR method, which is proposed for the EHP mutants’ investigation in epidemiological studies.

## Data availability statement

The data presented in the study are deposited in the NCBI GenBank repository, accession number MW269619.

## Author contributions

WF and HT designed the experiments. JZ and YW performed the experiments and prepared the manuscript. MY, NY, and YX participated in molecular analyses. WL and XL constructed the figures. JY assisted in sample collection. All authors contributed to the article and approved the submitted version.

## Funding

The research was supported by Fundamental Research Funds for Public Research Institutes at central level (East China Sea Fisheries Research Institute) (No.2019M03) and the Central Public-interest Scientific Institution Basal Research Fund, CAFS (No. 2020TD41).

## Acknowledgments

The authors acknowledge Liwen Xu for providing *Enterospora epinepheli*, and Hongbo Jiang for providing *Enterocytospora artemiae* used in this research.

## Conflict of interest

The authors declare that the research was conducted in the absence of any commercial or financial relationships that could be construed as a potential conflict of interest.

## Publisher’s note

All claims expressed in this article are solely those of the authors and do not necessarily represent those of their affiliated organizations, or those of the publisher, the editors and the reviewers. Any product that may be evaluated in this article, or claim that may be made by its manufacturer, is not guaranteed or endorsed by the publisher.
